# Airway Inflammation in Chronic Rhinosinusitis with Nasal Polyps and Asthma: The United Airways Concept Further Supported

**DOI:** 10.1371/journal.pone.0127228

**Published:** 2015-07-01

**Authors:** Kåre Håkansson, Claus Bachert, Lars Konge, Simon Francis Thomsen, Anders Elm Pedersen, Steen Seier Poulsen, Tomas Martin-Bertelsen, Ole Winther, Vibeke Backer, Christian von Buchwald

**Affiliations:** 1 Department of Otorhinolaryngology, Head & Neck Surgery and Audiology, Rigshospitalet, University of Copenhagen, Copenhagen, Denmark; 2 Upper Airways Research Laboratory, Ghent University Hospital, Ghent, Belgium, ENT-Department, Karolinska Institute, Stockholm, Sweden; 3 Centre for Clinical Education, University of Copenhagen and the Capital Region of Denmark, Copenhagen, Denmark; 4 Department of Respiratory Medicine L, Bispebjerg Hospital, University of Copenhagen, Copenhagen, Denmark; 5 Department of International Health, Immunology and Microbiology, University of Copenhagen, Copenhagen, Denmark; 6 Department of Biomedical Sciences, Endocrinology Research Section, University of Copenhagen, Copenhagen, Denmark; 7 The Bioinformatics Centre (BINF), Department of Biology and Biotech Research and Innovation Centre (BRIC), University of Copenhagen, Copenhagen, Denmark; 8 Section for Cognitive Systems, DTU Compute Technical University of Denmark (DTU), Copenhagen, Denmark; University of Tennessee Health Science Center, UNITED STATES

## Abstract

**Background:**

It has been established that patients with chronic rhinosinusitis with nasal polyps (CRSwNP) often have co-existing asthma.

**Objective:**

We aimed to test two hypotheses: (i) upper and lower airway inflammation in CRSwNP is uniform in agreement with the united airways concept; and (ii) bronchial inflammation exists in all CRSwNP patients irrespective of clinical asthma status.

**Methods:**

We collected biopsies from nasal polyps, inferior turbinates and bronchi of 27 CRSwNP patients and 6 controls. All participants were evaluated for lower airway disease according to international guidelines. Inflammatory cytokines were investigated using a Th1/Th2 assay including 14 chemokines and cytokines; tissue concentrations were normalized according to tissue weight and total protein concentration. Individual cytokines and multivariate inflammatory profiles were compared between biopsy sites and between patients and controls.

**Results:**

We found significantly higher concentrations of Th2 cytokines in nasal polyps compared to inferior turbinate and bronchial biopsies. In addition, we showed that the inflammatory profile of nasal polyps and bronchial biopsies correlated significantly (p<0.01). From the Th2 cytokines measured, IL-13 was significantly increased in bronchial biopsies from CRSwNP patients with, but not without asthma.

**Conclusion:**

Our findings support the united airways concept; however, we did not find evidence for subclinical bronchial inflammation in CRSwNP patients without asthma. Finally, this study indicates for the first time that nasal polyps potentially play an important role in the airway inflammation rather than being a secondary phenomenon.

## Introduction

It has been reported that 20–60% of patients with chronic rhinosinusitis with nasal polyps (CRSwNP) have asthma. [1–3] However, the pathogenetic mechanism leading to CRSwNP is uncertain; likewise, it is unknown why so many CRSwNP patients develop co-existing asthma. It is assumed that the inflammatory profile is similar throughout the airway in CRSwNP patients with co-existing asthma. However, before the present study, evidence for this assumption was sparse and primarily derived from independent studies of the upper airway in CRS patients and the lower airways in asthma patients.

Outside China, CRSwNP is an eosinophilic disease characterized by Th2 cytokines such as interleukin (IL)-5, eotaxin and IL-13. [2,4,5] Nasal polyps from patients with asthma, compared to those without, present with a stronger degree of eosinophilic inflammation. [6–10] Furthermore, higher concentrations of IL-5, IL-9 and eotaxin and more eosinophils and lymphocytes have been shown in bronchial biopsies and lavages from CRSwNP patients with asthma or airway hyperresponsiveness (AHR) compared with CRSwNP patients without asthma or AHR. [11–13] So far, only one study has compared nasal and bronchial inflammation in the same CRSwNP patients. [14] Ragab et al. compared the cytology of nasal and bronchial lavages in patients with CRS and found no correlation; however, both CRS with and without nasal polyps were included in the study. Ediger et al. analyzed biopsies from polyps, inferior turbinates (ITs) and bronchii using immunohistochemistry in CRSwNP patients with asthma, AHR and patients without lower airway disease. [15] Only small differences were found between groups—possibly because only non-steroid dependent asthma patients were included—and more importantly, no paired analysis of nasal vs. bronchial biopsies was performed. None of the above studies included bronchial biopsies from controls and to date, no study has compared the inflammatory profile in upper and lower airway biopsies in the same population of CRSwNP patients.

We here hypothesized that (i) upper and lower airway inflammation in CRSwNP show similar profiles indicating a common pathogenic mechanism. We further hypothesized that (ii) bronchial inflammation is present in all CRSwNP patients irrespective of their clinical asthma status.

## Materials and Methods

### Design

Patients who met the European Position Paper on Rhinosinusitis and Nasal Polyps (EP^3^OS) criteria for CRSwNP [16] and agreed to participate were consecutively included during June 2011–January 2013 at the Department of Otorhinolaryngology, Head and Neck Surgery and Audiology, Rigshospitalet. All patients lived in or around Copenhagen and were referred for functional endoscopic sinus surgery (FESS); patients were included irrespectively of former surgery. All had been treated according to the EP^3^OS guideline with nasal steroids and saline irrigations for more than three months without recovery. Topical nasal steroids, but not inhaled steroids, were discontinued four weeks before surgery. A control group comprised non-asthmatic patients undergoing nasal septal surgery and/or turbinate reduction. Lower airway disease was classified according to the Global Initiative for Asthma (GINA) and Global initiative for chronic Obstructive Lung Disease (GOLD) guidelines. [17,18] For details about the clinical evaluation, please see previous publication by Håkansson et al. [19]

Exclusion criteria were: age below 18 or above 80 years; systemic steroids within 3 months before inclusion; severe psychiatric disorders; need for interpreter; pregnancy or nursing; immunodeficiencies; chronic obstructive pulmonary disease (COPD), sarcoidosis, cystic fibrosis, primary ciliary dyskinesia; systemic vasculitis; unstable cardiovascular disease; dysregulated diabetes, forced expiratory volume in the 1^st^ second (FEV1) < 0.5 liter; trombocytopenia or international normalized ratio (INR) > 1.5.

#### Ethics statement

The study was conducted in accordance with the Declaration of Helsinki and was approved by the Ethics Committee of Copenhagen County (H-B-2008-106); a written informed consent was obtained from all participants.

#### Biopsies and BAL

We collected biopsies from nasal polyps, ITs and bronchii of 27 CRSwNP patients and 6 controls. IT biopsies were unavailable from seven of the CRSwNP patients. All patients and controls were of European or Western Asian descent. For patient characteristics, see Table 1.

**Table 1:** Demographics for the biopsy groups.

	CRSwNP +asthma n = 18	CRSwNP–asthma n = 9	Controls n = 6
Age (median)	46 (25–70)	55 (27–68)	35 (22–54)
Sex (male) %	67	67	83
Atopy %	44	33	0
Inhaled steroids %	61	0	0
Samter’s triad %	17	0	0

CRSwNP = chronic rhinosinusitis with nasal polyps.

During FESS, nasal biopsies (polyp and ITs) were taken endoscopically. Punch biopsies from the IT were taken >2 cm posteriorly to its anterior edge. Subsequently, bronchoscopy was performed and 6–8 biopsies were taken from the subcarinae of the right inferior lobe according to guidelines.[20] Biopsies were snap frozen (in the operating room) in liquid nitrogen and kept at -80°C until homogenization. Large biopsies were cut into smaller pieces to preserve primarily the superficial layers.

Biopsies were homogenized using a Tissuelyser LT (Qiagen, Hilden, Germany). Samples were precooled in liquid nitrogen for at least 30 minutes and then disrupted and homogenized without lysis buffer. One ml of 0.9% NaCl + Protease Inhibitor per 0.1 g tissue (Compete # 1 697 498; Roche Diagnostics) was added after disruption and homogenization. Samples were centrifuged at 3000 rpm for 10 min at 4°C and aliquots were kept at -80°C until analysis. From tissue homogenates, the protein concentration was determined using a standard BCA assay and the measured values were normalized to a concentration of 100 μg total protein per sample (in 25 μl pr well), based on parallel analysis of protein concentrations. Homogenates were analyzed using an ultra-sensitive human chemokine 7-plex assay (cat# K15031C-2) and an ultra-sensitive human Th1/Th2 cytokine assay (cat# K15011-C2, Mesoscale Diagnostics, LCC, Rockwill, US), according to the protocol from the manufacturer. Included biomarkers were: eotaxin, interleukin (IL)-8, interferon-inducable protein (IP)-10, monocyte chemotactic protein (MCP)-1, MCP-4, macrophage inflammatory protein (MIP)-1β, thymus- and activation-regulated chemokine (TARC), interferon (INF)-γ, IL-10, IL-12p70, IL-13, IL-2, IL-4, IL-5 (for BAL, only the chemokine assay was performed). All plates were analyzed using a Sector Imager from Mesoscale Diagnostics, LCC.

### Statistics

In CRSwNP patients outside China, IL-5 has been shown to be a defining cytokine in CRSwNP; therefore Spearman’s rho was used to identify IL-5-correlated cytokines (Spearman’s rho >0.6 and p<0.001) within nasal polyps. These cytokines were used in the subsequent between-group analysis using the Kruskal-Wallis test. A paired Wilcoxon Signed Rank test was applied for comparison of cytokine concentrations at different airway levels. Furthermore, to increase comparability with previous studies, Spearman’s rho was used for a second analysis of the cytokine correlation between biopsy sites (nasal polyps vs. bronchii).

To prepare data for multivariate analysis, biomarker concentrations were log transformed producing roughly normally distributed data. An exploratory principal component analysis (PCA) on the data from both locations revealed location as the main source of variation. Therefore in order to at least partially remove this source of variation, the log-transformed data were standardized (mean subtracted and divided by standard deviation) for each location separately. The exploratory PCA and bi-plot analysis also showed a high degree of co-variation within the IL-5 correlated cytokines (Th2) and the remaining cytokines (mainly Th1) further supporting that we could treat these as two groups (profiles) in the multivariate analysis (Th1 and Th2).

A low Euclidian distance between the patient’s transformed cytokine levels in upper (nasal polyps or ITs) and lower airway biopsies indicates that the cytokine signal co-varied between the upper and lower airways. We devised a permutation test where the average patient Euclidian distance was compared to the average distance calculated by randomly pairing measurements from the two locations in different patients. [21] The null hypothesis was that the distance between cytokine levels at the two locations was not patient specific. A p-value of 0.05 meant that 5% of the average permutation distances came out lower than the observed averaged distance in the patients.

R was used for the PCA and permutation test [22] and SPSS version 19.0 (IBM, Chicago, IL) was used for all other analyses; a p-value < 0.05 was considered statistically significant.

## Results

**Fig 1:**
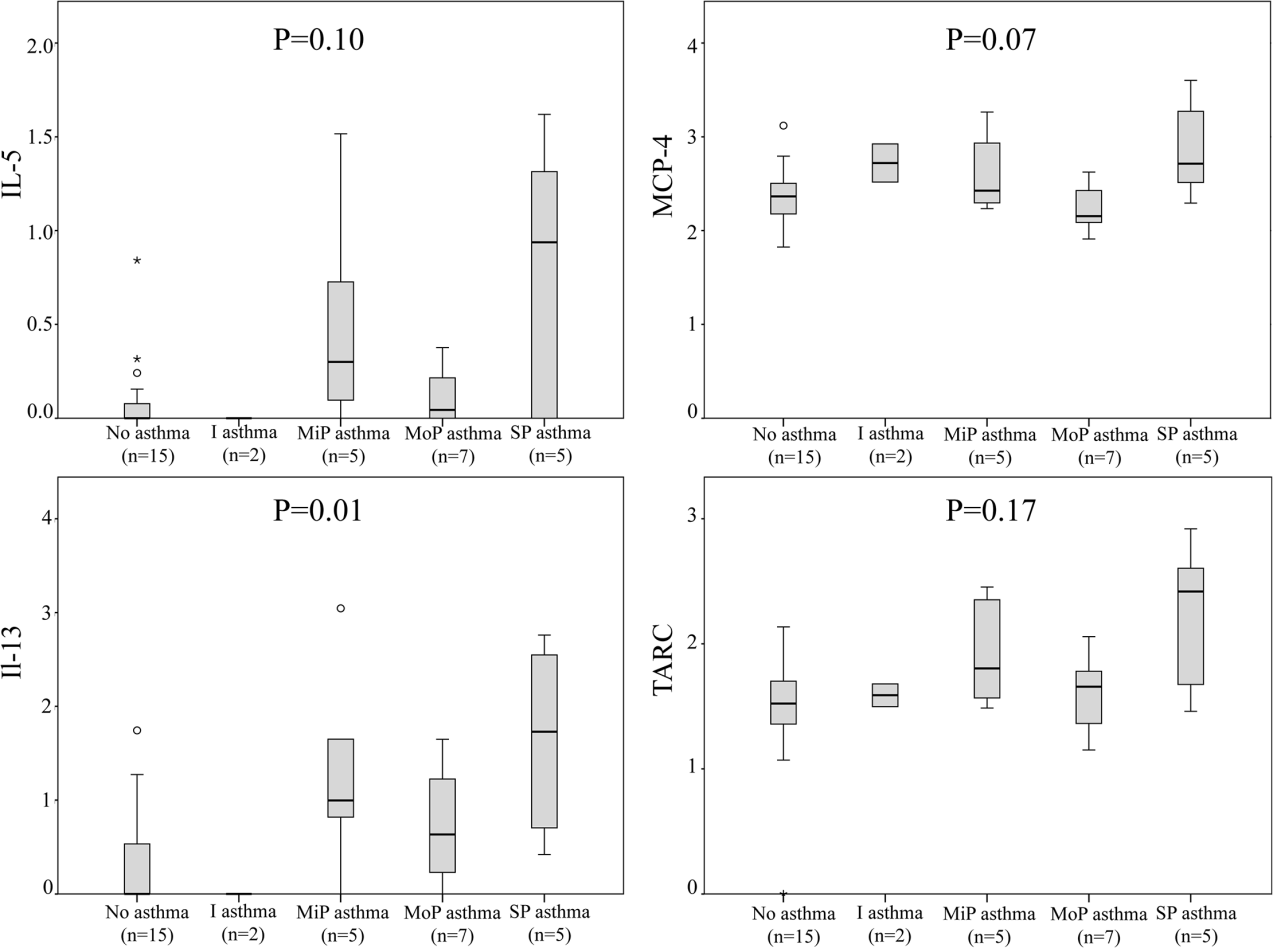
Inflammatory cytokines in bronchial biopsies (MCP-4, IL-5, IL-13 and TARC) in CRSwNP patients and controls, stratified by asthma severity. Log transformed data, concentrations in pg/mL. I = Intermittent; MiP = Mild persistent; MoP = Moderate persistent; SP = Severe persistent. P-values represent comparison by Kruskal-Wallis test. In this analysis we included biopsies from controls with asthma that were not included in the inter-group analyses. This analysis indicates that bronchial inflammation increases with increasing asthma-severity.

Demographic data are reported in Table 1. In nasal polyps, IL-5 correlated highly with other Th2 cytokines: eotaxin, MCP-1, MCP-4, TARC, IL-4 and IL-13. Univariate analysis of these cytokines failed to show bronchial inflammation in CRSwNP patients without asthma (Table 2); however, in patients with asthma, an increased IL-13 indicated bronchial inflammation in biopsies. A trend towards higher bronchial concentrations of Th2 cytokines was seen with increasing asthma severity (Fig 1).

**Table 2:** Comparison of cytokines in CRSwNP with/without asthma and controls.

	CRSwNP +asthma	CRSwNP–asthma	Controls		
Bronchial biopsies (pg/ml)	n = 18	n = 9	n = 6	P	P adjusted
Eotaxin	603 (114–1828)	535 (143–1086)	479 (174–719)	0.47	-
MCP-1	278 (88–2151)	341 (301–906)	258 (148–1234)	0.55	-
MCP-4	281 (80–3999)	289(72–1316)	193 (66–256)	0.27	-
TARC	47 (13–830)	45 (0–136)	22 (11–52)	0.06	-
IL-13	7 (0–1108)	0 (0–54)	1 (0–3.47)	0.04	0.05*
IL-4	0 (0–5)	0 (0–0)	0.03 (0–0.32)	0.11	-
IL-5	0.18 (0–41)	0 (0–6)	0 (0–0.43)	0.13	-

*Between the CRSwNP with and without asthma groups

CRSwNP = chronic rhinosinusitis with nasal polyps. NS = non-significant. Median concentrations of biomarkers in pg/ml (min-max). P-values calculated by Kruskal-Wallis. Due to multiple comparisons, Bonferroni-adjusted p-values are reported.

**Fig 2:**
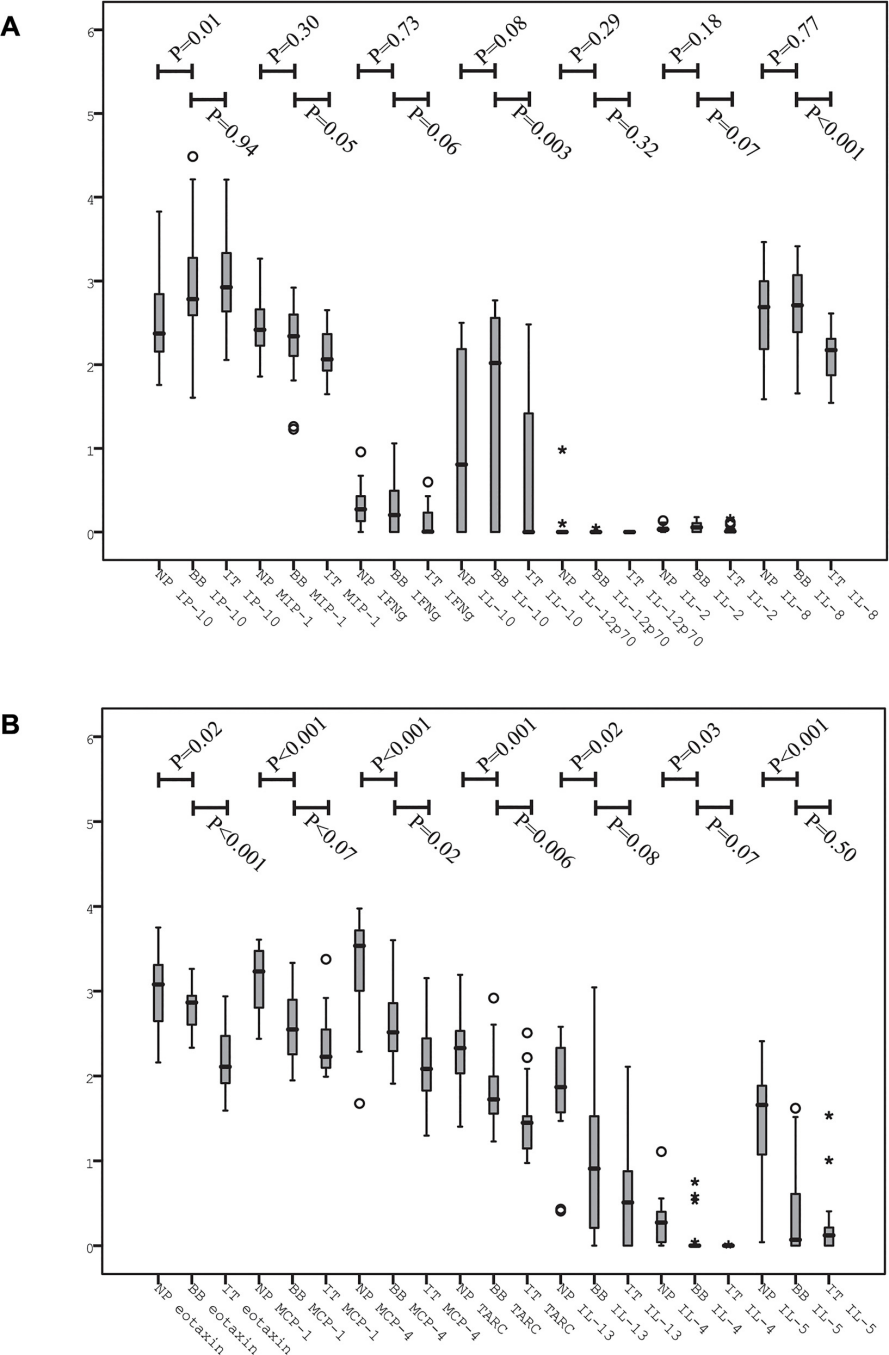
Concentration of Th1 and Th2 cytokines in nasal polyps, ITs and bronchial biopsies in CRSwNP patients. A: Th1 cytokines. B: Th2 cytokines. Log transformed data, concentrations in pg/mL. NP = nasal polyp, BB = bronchial biopsy, IT = inferior turbinate. P-values represent comparison by a paired Wilcoxon signed rank test. Both CRSwNP patients with and without asthma are included in this analysis. All Th2 cytokines were increased in the nasal polyps compared to the bronchii; this difference was not seen for Th1 cytokines.

After normalization for tissue weight and total protein concentration, we found significantly higher concentrations of Th2 cytokines in nasal polyp biopsies in comparison with bronchial biopsies (p<0.05 for all); however, lower Th2 cytokine concentrations were found at the level of the IT (Fig 2B). Most of the remaining cytokines (mainly Th1) did not differ significantly between nasal polyps and bronchial biopsies except for IP-10 that was significantly decreased in nasal polyps.

When applying a univariate correlation analysis, only IL-4 correlated significantly between nasal polyp and bronchial biopsies; however, this was due to three outliers (Table 3).

**Table 3:** Correlation individual cytokines in the nasal polyps and bronchial biopsies.

Cytokine	Spearman’s rho	P
Eotaxin	0.11	0.6
MCP-1	0.22	0.26
MCP-4	0.37	0.06
TARC	0.30	0.12
IL-13	0.30	0.12
IL-4	0.40	0.04
IL-5	0.17	0.40

Two-sided p-values are reported.

An insignificant correlation is seen for several cytokines. Only IL-4 was significantly correlated, but this correlation was driven by 3 outliers.

Finally, using the permutation test we rejected the null hypothesis of no patient specific association between nasal polyp and bronchial inflammation (p = 0.0029 and p = 0.0063 for Th1 and Th2 cytokines, respectively). The correlation between IT and bronchial inflammation was less consistent in patients (p<0.001 and p = 0.15 for Th1 and Th2 cytokines, respectively) and controls (p = 0.12 and p = 0.01 for Th1 and Th2 cytokines, respectively).

## Discussion

This is the first study that simultaneously investigates inflammatory cytokines in biopsies from the upper and lower airways in the same CRSwNP patients; in addition, this is the first study to compare bronchial inflammation in CRSwNP patients with controls. In CRSwNP patients, we found higher concentrations of Th2 cytokines in nasal polyps compared with bronchial biopsies and ITs and a strong correlation between the upper and lower airway inflammatory profiles was seen. None of the measured cytokines were significantly increased in the bronchi of CRSwNP patients except for IL-13 in CRSwNP patients with asthma. Taken together, these findings indicate that a similar inflammation exists throughout the airways in CRSwNP patients and that nasal polyps might play an important role in this airway inflammation. Our findings could not confirm the presence of subclinical bronchial inflammation in CRSwNP patients without asthma.

### Comparing inflammation throughout the airways

It has been hypothesized that asthma simply develops in allergic airway disease when the inflammation of the upper airway is expressive enough—a hypothesis that is also feasible in the context of CRSwNP and asthma. [23] Studies have shown a positive association between asthma and the degree of nasal polyp inflammation [6–10,24,25], which potentially explains why asthma is associated with a poorer clinical outcome in CRSwNP patients. [26,27] In addition, a stronger bronchial inflammation has been shown in CRSwNP patients with asthma compared to those without. [11–13] However, one study, based on immunohistochemistry, did not confirm this relationship; this study instead reported that the airway inflammation in CRSwNP patients was stronger in the nasal polyps, and then faded towards the bronchii. [15] In line with this finding, we found higher concentrations of Th2 inflammatory cytokines in nasal polyps compared to bronchial biopsies and ITs in CRSwNP patients.

Our study thus indicates that nasal polyps might be an epicenter of airway inflammation in patients with CRSwNP and asthma. However, the decreased inflammatory response of the bronchii and ITs could also have resulted from a hypothetical protective mechanism outside the nasal polyps—a mechanism that might also explain why polyps normally are absent at the level of the ITs and bronchii. Furthermore, our findings might be ascribed to selection bias: as patients are included from the department of otorhinolaryngology, patients with severe upper airway inflammation are selected over those with primarily lower airway inflammation. Finally, we found a trend towards higher bronchial cytokine concentrations with increasing asthma severity indicating that had we included cases with more severe asthma, stronger bronchial inflammation would have been reported (Fig 1).

### The correlation of inflammatory profiles

We compared biopsies from three different levels of the airway: nasal polyps, ITs and bronchii. We assumed that the inflammation was comparable across biopsy sites since the entire airway is lined with respiratory pseudostratified epithelium with underlying lamina propria; however, organ specific structures are situated just below these layers such as glands, vascular structures and smooth muscle. We took this heterogeneity between biopsy sites into consideration during biopsy sampling by preserving primarily the superficial layers before snap freezing; thereby we removed most of the organ specific structures while preserving the inflammatory cells that were situated superficially. [7] In addition, during the post-hoc correlation analysis, we standardized data to adjust for some of the differences between biopsy sites.

It has previously been assumed that a similar inflammatory profile exist throughout the airway in CRSwNP patients with co-existing asthma. However, before the present study, evidence for this assumption was sparse and primarily derived from independent studies of the upper airway in CRS patients and the lower airways in asthma patients. Only Ragab et al. had compared upper and lower airway inflammation in the same patients—and failed to show a correlation. [14] When we applied the same statistical method as Ragab et al., no unequivocal correlation of individual cytokines was found (Table 3). However, when using a permutation analysis that allowed us to analyze the correlation of Th1 and Th2 cytokine profiles instead of individual cytokines, a significant interrelation was found. This approach revealed a strong correlation between nasal polyp and bronchial inflammation indicating that specificity increased when analyzing individual patient’s inflammation profiles. A correlation was also found between IT and bronchial biopsies, both in patients and controls; however, this correlation did not apply for both Th1 and Th2 cytokines. This demonstrates that even in the non-inflamed airway, the profile of inflammatory cytokines is similar throughout the airway. Since the inflammation in the nasal polyps was clearly Th2 driven, the strong correlation reported here indicates that the bronchial inflammation in CRSwNP patients, however insignificant, also was Th2 skewed.

### Comparing inflammation between groups

Subclinical upper and lower airway inflammation has been shown in allergic airway disease. [28] In continuation of these findings, we hypothesized that all patients with CRSwNP have bronchial inflammation irrespective of the asthma status. However, we could not confirm subclinical inflammation in bronchial biopsies from CRSwNP patients without asthma. Even in CRSwNP patients with asthma, bronchial inflammation was ambiguous and characterized mainly by higher bronchial IL-13. Potentially, a more sensitive analysis could have shown bronchial inflammation irrespective of asthma. Furthermore, we speculate that a trend for higher concentrations was seen for several cytokines, indicating that a larger sample size could have changed our conclusion. Thus, we might be at risk of a type II error when rejecting the (ii) hypothesis.

### Limitations

We found lower concentrations of Th2 cytokines in bronchial biopsies when compared to nasal polyps—with and without standardization for total protein. This could in part be explained by the continuation of inhaled steroids in 60% of the asthmatic CRSwNP patients, whereas nasal steroids were discontinued four weeks before the operation [29–31]; alternatively it could be due to the inclusion of both CRSwNP patients with and without asthma into the analysis. However, Th1 cytokines were not depressed which in part contradicts a significant effect of inhaled steroids. [32] Secondly, an analysis of CRSwNP patients who did not take inhaled steroids still found that 5/7 Th2 cytokines were significantly increased in the nasal polyps compared to the bronchii (MCP-1, MCP-4, TARC, IL-13 and IL-5, S1 Table). Thirdly, bronchial cytokine concentrations were independent of inhaled steroids in the asthmatic CRSwNP group (S2 Table) and except for IL-13, no significant difference in bronchial inflammations was found between asthmatic and non-asthmatic CRSwNP patients (Table 2).

Finally, an important limitation was the poor match of atopy between groups and the inclusion of three patients with Samter’s triad. However, when analysing non-atopics only or when excluding the three patients with Samter’s triad, the conclusions from our finding remained unchanged.

In conclusion, we found higher Th2 cytokine concentrations in nasal polyps compared with the remaining airway and a strong correlation between the upper and lower airway inflammatory profiles was seen; however, we could not confirm the presence of subclinical bronchial inflammation in CRSwNP patients without asthma. Our findings indicate that a similar inflammation exists throughout the airways in CRSwNP patients and potentially nasal polyps play an important role in this airway inflammation rather than being a secondary phenomenon.

## Supporting Information

S1 Table Difference in Th2 cytokine concentrations in nasal polyps and bronchial biopsies. In CRSwNP patients who do not take inhalation steroids. P-values represent comparison by a paired Wilcoxon signed rank test.
(DOCX)

S2 Table Difference in bronchial cytokine concentrations with and without inhalation steroid usage. Astmatic CRSwNP patients only. P-values represent comparison by a paired Wilcoxon signed rank test.
(DOCX)

S1 Dataset Measured concentrations of 14 Th1/Th2 cytokines in NP, IT and bronchial biopsies. Concentrations in pg/ml.
(SAV)

## References

[1] Larsen K. The clinical relationship of nasal polyps to asthma. Allergy Asthma Proc; 17(5):243–9. 8922143 10.2500/108854196778662255

[2] Zhang N, Van Zele T, Perez-Novo C, Van Bruaene N., Holtappels G, DeRuyck N, et al Different types of T-effector cells orchestrate mucosal inflammation in chronic sinus disease. J Allergy Clin Immunol; 122(5):961–8. 10.1016/j.jaci.2008.07.008 18804271

[3] Klossek JM, Neukirch F, Pribil C, Jankowski R, Serrano E, Chanal I, et al Prevalence of nasal polyposis in France: a cross-sectional, case-control study. Allergy; 60(2):233–7. 15647046 10.1111/j.1398-9995.2005.00688.x

[4] Granstrom G, Jacobsson E, Jeppsson PH. Influence of allergy, asthma and hypertension on nasal polyposis. Acta Otolaryngol Suppl; 492:22–7. 1632246 10.3109/00016489209136803

[5] Bachert C, Zhang N. Chronic rhinosinusitis and asthma: novel understanding of the role of IgE 'above atopy'. J Intern Med; 272(2):133–43. 10.1111/j.1365-2796.2012.02559.x 22640264

[6] Ardehali MM, Amali A, Bakhshaee M, Madani Z, Amiri M. The comparison of histopathological characteristics of polyps in asthmatic and nonasthmatic patients. Otolaryngol Head Neck Surg; 140(5):748–51. 10.1016/j.otohns.2009.01.027 19393423

[7] Bateman ND, Shahi A, Feeley KM, Woolford TJ. Activated eosinophils in nasal polyps: a comparison of asthmatic and non-asthmatic patients. Clin Otolaryngol; 30(3):221–5. 16111416 10.1111/j.1365-2273.2005.00969.x

[8] Dhong HJ, Kim HY, Cho DY. Histopathologic characteristics of chronic sinusitis with bronchial asthma. Acta Otolaryngol; 125(2):169–76. 15880948 10.1080/00016480410015767

[9] Haruna S, Nakanishi M, Otori N, Moriyama H. Histopathological features of nasal polyps with asthma association: an immunohistochemical study. Am J Rhinol; 18(3):165–72. 15283491

[10] Bachert C, Zhang N, Holtappels G, De Lobel L., van Cauwenberge P., Liu S, et al Presence of IL-5 protein and IgE antibodies to staphylococcal enterotoxins in nasal polyps is associated with comorbid asthma. J Allergy Clin Immunol; 126(5):962–8, 968. 10.1016/j.jaci.2010.07.007 20810157

[11] Tsicopoulos A, Shimbara A, de Nadai P., Aldewachi O, Lamblin C, Lassalle P, et al Involvement of IL-9 in the bronchial phenotype of patients with nasal polyposis. J Allergy Clin Immunol; 113(3):462–9. 15007348 10.1016/j.jaci.2003.12.009

[12] Lamblin C, Bolard F, Gosset P, Tsicopoulos A, Perez T, Darras J, et al Bronchial interleukin-5 and eotaxin expression in nasal polyposis. Relationship with (a)symptomatic bronchial hyperresponsiveness. Am J Respir Crit Care Med; 163(5):1226–32. 11316663 10.1164/ajrccm.163.5.2004197

[13] Lamblin C, Gosset P, Salez F, Vandezande LM, Perez T, Darras J, et al Eosinophilic airway inflammation in nasal polyposis. J Allergy Clin Immunol; 104(1):85–92. 10400844 10.1016/s0091-6749(99)70118-1

[14] Ragab A, Clement P, Vincken W. Correlation between the cytology of the nasal middle meatus and BAL in chronic rhinosinusitis. Rhinology; 43(1):11–7. 15844496

[15] Ediger D, Sin BA, Heper A, Anadolu Y, Misirligil Z. Airway inflammation in nasal polyposis: immunopathological aspects of relation to asthma. Clin Exp Allergy; 35(3):319–26. 15784110 10.1111/j.1365-2222.2005.02194.x

[16] Fokkens WJ, Lund VJ, Mullol J, Bachert C, Alobid I, Baroody F, et al European Position Paper on Rhinosinusitis and Nasal Polyps 2012. Rhinol Suppl; (23):3–298. 22764607

[17] Fabbri L, Pauwels RA, Hurd SS. Global Strategy for the Diagnosis, Management, and Prevention of Chronic Obstructive Pulmonary Disease: GOLD Executive Summary updated 2003. COPD; 1(1):105–41. 16997745 10.1081/COPD-120030163

[18] Bateman ED, Hurd SS, Barnes PJ, Bousquet J, Drazen JM, FitzGerald M, et al Global strategy for asthma management and prevention: GINA executive summary. Eur Respir J; 31(1):143–78. 10.1183/09031936.00138707 18166595

[19] Håkansson K, Thomsen SF, Konge L, Mortensen J, Backer V, von Buchwald C. A comparative and descriptive study of asthma in chronic rhinosinusitis with nasal polyps. American Journal of Rhinology & Allergy; 28(5):383–7. 10.2500/ajra.2014.28.4076 25198023

[20] British Thoracic Society Bronchoscopy Guidelines Committee. British Thoracic Society guidelines on diagnostic flexible bronchoscopy. Thorax; 56 Suppl 1:i1–21. 11158709 10.1136/thorax.56.suppl_1.i1 PMC1765978

[21] Good PI. Permutation, Parametric, and Bootstrap Tests of Hypotheses (Springer Series in Statistics) Springer; 2005.

[22] Team R. R Development Core Team 2013. R: A Language and Environment for Statistical Computing.

[23] Togias A. Rhinitis and asthma: evidence for respiratory system integration. J Allergy Clin Immunol; 111(6):1171–83. 12789212 10.1067/mai.2003.1592

[24] Staikuniene J, Vaitkus S, Japertiene LM, Ryskiene S. Association of chronic rhinosinusitis with nasal polyps and asthma: clinical and radiological features, allergy and inflammation markers. Medicina (Kaunas); 44(4):257–65. 18469501

[25] Han DH, Kim SW, Cho SH, Kim DY, Lee CH, Kim SS, et al Predictors of bronchial hyperresponsiveness in chronic rhinosinusitis with nasal polyp. Allergy; 64(1):118–22. 10.1111/j.1398-9995.2008.01841.x 19120071

[26] Larsen K, Tos M. A long-term follow-up study of nasal polyp patients after simple polypectomies. Eur Arch Otorhinolaryngol; 254 Suppl 1:S85–S88. 9065636 10.1007/BF02439732

[27] Larsen K, Tos M. Clinical course of patients with primary nasal polyps. Acta Otolaryngol; 114(5):556–9. 7825441 10.3109/00016489409126104

[28] Braunstahl GJ, Fokkens W. Nasal involvement in allergic asthma. Allergy; 58(12):1235–43. 14616096 10.1046/j.0105-4538.2003.00354.x

[29] Bachert C, Gevaert P, Holtappels G, Cuvelier C, van Cauwenberge P. Nasal polyposis: from cytokines to growth. Am J Rhinol; 14(5):279–90. 11068652 10.2500/105065800781329573

[30] Kanai N, Denburg J, Jordana M, Dolovich J. Nasal polyp inflammation. Effect of topical nasal steroid. Am J Respir Crit Care Med; 150(4):1094–100. 7921442 10.1164/ajrccm.150.4.7921442

[31] Wallwork B, Coman W, Feron F, Mackay-Sim A, Cervin A. Clarithromycin and prednisolone inhibit cytokine production in chronic rhinosinusitis. Laryngoscope; 112(10):1827–30. 12368623 10.1097/00005537-200210000-00022

[32] Snijdewint FG, Kapsenberg ML, Wauben-Penris PJ, Bos JD. Corticosteroids class-dependently inhibit in vitro Th1- and Th2-type cytokine production. Immunopharmacology; 29(2):93–101. 7775161 10.1016/0162-3109(94)00048-k

