# Supplementary material for: Airway Inflammation in Chronic Rhinosinusitis with Nasal Polyps and Asthma: The United Airways Concept Further Supported
Source: PLoS One. 2015 Jul 1;10(7):e0127228. doi: 10.1371/journal.pone.0127228 (PMC4489400; doi:10.1371/journal.pone.0127228)
Supplement: S1 Table — In CRSwNP patients who do not take inhalation steroids. P-values represent comparison by a paired Wilcoxon signed rank test. (DOCX) [file pone.0127228.s001.docx]

| Cytokine | p |
| --- | --- |
| Eotaxin | 0.09 |
| MCP-1 | 0.002 |
| MCP-4 | 0.002 |
| TARC | 0.008 |
| IL-13 | 0.03 |
| IL-4 | 0.07 |
| IL-5 | 0.02 |

S1 Table. Difference in Th2 cytokine concentrations in nasal polyps and bronchial biopsies.

In CRSwNP patients who do not take inhalation steroids. P-values represent comparison by a paired Wilcoxon signed rank test
