# Supplementary material for: Airway Inflammation in Chronic Rhinosinusitis with Nasal Polyps and Asthma: The United Airways Concept Further Supported
Source: PLoS One. 2015 Jul 1;10(7):e0127228. doi: 10.1371/journal.pone.0127228 (PMC4489400; doi:10.1371/journal.pone.0127228)
Supplement: S2 Table — Astmatic CRSwNP patients only. P-values represent comparison by a paired Wilcoxon signed rank test. (DOCX) [file pone.0127228.s002.docx]

| Cytokine | p |
| --- | --- |
| Eotaxin | 0.86 |
| MCP-1 | 0.25 |
| MCP-4 | 0.18 |
| TARC | 0.38 |
| IL-13 | 0.21 |
| IL-4 | 0.66 |
| IL-5 | 0.25 |

S2 Table. Difference in bronchial cytokine concentrations with and without inhalation steroid usage

Astmatic CRSwNP patients only. P-values represent comparison by a paired Wilcoxon signed rank test
